# Surveillance System for Infectious Diseases of Pets, Santiago, Chile

**DOI:** 10.3201/eid1510.081596

**Published:** 2009-10

**Authors:** Javier López, Katia Abarca, Jaime Cerda, Berta Valenzuela, Lilia Lorca, Andrea Olea, Ximena Aguilera

**Affiliations:** Chilean Society of Veterinary Infectious Diseases, Santiago, Chile (J. López, B. Valenzuela, L. Lorca); Pontificia Universidad Católica de Chile, Santiago (K. Abarca, J. Cerda); Ministry of Health, Santiago (A. Olea, X. Aguilera)

**Keywords:** Sentinel surveillance, pets, domestic animals, communicable diseases, bacteria, viruses, parasites, dispatch

## Abstract

Pet diseases may pose risks to human health but are rarely included in surveillance systems. A pilot surveillance system of pet infectious diseases in Santiago, Chile, found that 4 canine and 3 feline diseases accounted for 90.1% and 98.4% of notifications, respectively. Data also suggested association between poverty and pet diseases.

**Communicable diseases challenge health systems and require coordinated efforts for their control. Surveillance systems for human communicable diseases have been implemented since the 19th century. Surveillance of animal infections started later and focused on livestock production. More recently, in response to emerging zoonoses such as avian influenza and West Nile virus infection, novel surveillance systems for wild animals have been implemented (*1*). Although pet-borne infections have become increasingly relevant to human health, systematic notification of these infections is not currently conducted, except for rabies.**

**Pets (domesticated dogs and cats that live in close proximity to humans) may pose several risks to their owners’ health and create occupational hazards for professionals such as veterinarians. They can also serve as sentinels for several diseases by alerting persons to the presence of infectious agents in a community (*2*). These features emphasize the need for surveillance systems of pet infectious diseases, especially those that can be transmitted to humans (*3*).**

In Chile, as in other countries, pet infectious diseases, except for rabies, have not been included in any surveillance system; for this reason, information about their epidemiology is scarce. Thus, a pilot surveillance system for infectious disease of pet dogs and cats was implemented for a 2-year period in Santiago, Chile.

## The Study

During October 2004–September 2006, the sentinel surveillance system was implemented in 61 veterinary clinics (30 during the first year and another 31 during the second year) located in 34 districts of Santiago (population 5.4 million). Pet population estimates (1,117,192 dogs; 518,613 cats) were derived from a study conducted previously in Santiago (*4*) and corresponded to a rate of ≈1 sentinel centers per 27,000 pets. Sentinel centers were asked to participate on a voluntary basis and were grouped similarly to the human health services, following geographic criteria.

Of the 12 notifiable infectious diseases in the surveillance system, 5 were nonzoonotic (distemper, canine infectious tracheobronchitis, feline respiratory complex disease, feline leukemia, and hemorrhagic gastroenteritis), and 7 were zoonotic (giardiasis, brucellosis, leptospirosis, rabies, ehrlichiosis, scabies, and tinea infection). Definitions were established for suspected and confirmed cases of each disease. Laboratory confirmation was required for diagnosis of giardiasis, brucellosis, leptospirosis, and rabies. Personnel from each sentinel center recorded their data on a website. They were trained in operative definitions and procedures, which included submitting a weekly report of the total number of cases seen. Participation in the study was voluntary; no funding or incentives were offered.

During the 2-year period, 8,167 cases were reported: 6,974 (85.4%) in dogs and 1,193 (14.6%) in cats. Of these dogs and cats, 4,415 (63.3%) and 730 (61.2%), respectively, were males. Also, 4,524 (64.9%) dogs and 503 (42.2%) cats were <1 year of age. Data submitted during the first year of surveillance accounted for 67.5% of canine and 66.7% of feline diseases notifications.

A negative correlation was found between the average number of notifications per sentinel center (ANC) and time (8 trimesters) for dogs (ρ –0.95, p<0.01) and cats (ρ –0.93, p<0.01). A positive correlation, although not statistically significant, was found between the average poverty rate of the districts located in each health service (*5*) and the ANC for dogs (ρ +0.77, p = 0.07) and cats (ρ +0.43, p = 0.40) (Table 1).

**Table 1:** Average number of notifications per sentinel center, according to health service, Santiago, Chile, October 2004–September 2005*

Health Service	Poverty (%)†	Average no. notifications	
		Dogs	Cats
South−East	13.7	160.1	20.1
West	13.2	349.7	52.3
South	11.7	189.6	36.4
North	9.5	143.8	22.6
Central	8.0	62.0	22.3
East	6.6	74.5	18.5

*First year of pilot surveillance system †Average poverty rate of the districts belonging to each health service.

During the 2-year surveillance period, 4 canine diseases (hemorrhagic gastroenteritis, distemper, scabies, and infectious tracheobronchitis) accounted for 90.1% of notifications, and 3 feline diseases (respiratory disease complex, feline leukemia, and tinea) accounted for 98.4% of notifications (Table 2). For each disease, ANC during the first year of surveillance was calculated for centers located in South–East Health Service (SEHS), which had the highest poverty rate of its districts, and East Health Service (EHS), which had the lowest. For canine diseases, the ratios of ANC for SEHS/ANC for EHS were 3.5 (scabies), 2.5 (distemper), 2.2 (hemorrhagic gastroenteritis), and 1.8 (infectious tracheobronchitis); for feline diseases, these ratios were 1.2 (respiratory disease complex), 1.1 (feline leukemia), and 0.7 (tinea).

**Table 2:** Diseases reported for dogs and cats, Santiago, Chile, October 2004−September 2006*

Disease	No. (%) cases
Dogs	
Hemorrhagic gastroenteritis	2,315 (33.2)
Distemper	1,461 (20.9)
Scabies	1,307 (18.7)
Infectious tracheobronchitis	1,207 (17.3)
Tinea	447 (6.4)
Ehrlichiosis	185 (2.7)
Giardiasis	38 (0.5)
Brucellosis	9 (0.1)
Leptospirosis	5 (0.1)
Cats	
Respiratory complex disease	826 (69.2)
Feline leukemia	222 (18.6)
Tinea	127 (10.6)
Scabies	14 (1.2)
Giardiasis	4 (0.3)
Total	8,167 (100.0)

*All health services, first and second year of surveillance.

For each of the 7 diseases, we calculated the following ratio: total no. notifications for the whole surveillance period for all sentinel centers for pets <1 year of age/total no. notifications for the whole surveillance period for all sentinel centers for pets >1 year of age. Diseases most commonly occurring in pets <1 year of age were hemorrhagic gastroenteritis (10.9) and distemper (3.1); on the contrary, diseases whose ratio favors pets >1 year of age were feline leukemia (0.17) and infectious tracheobronchitis (0.43). Scabies in dogs, feline respiratory disease complex, and tinea in cats had ratios of ≈1.00 (1.07, 0.96, and 0.92, respectively).

## Conclusions

This pilot surveillance system indicated that overall notifications predominated for pets with 3 characteristics: canine, male, and age <1 year. These characteristics partially reflect the species and sex distribution of pets in the city, as shown by a **household survey of 2,100 homes in 7 districts of Santiago during 2001, which showed that 55.7% of household pets were dogs (62.3% males, 16.0% <1 year of age), and 23.5% were cats (57.9% males, 15.0% <1 year of age) (M.A.** Daza, 2002, unpub. data). **However, according to our data, the** predominance of notifications for animals <1 year of age seems to represent a higher risk associated with being <1 year of age.

The finding that 4 canine and 3 feline diseases were most frequently reported may be useful in many settings, such as disease control prioritization and identification of topics of interest for investigation. From a human health perspective, 1 canine disease (scabies) was zoonotic and 2 others (hemorrhagic gastroenteritis and infectious tracheobronchitis) included zoonotic agents in their list of possible etiologies; thus, the information provided by the surveillance system is useful for human physicians and policy makers. This finding is especially relevant because certain pet diseases may occur on a socioeconomic gradient, affecting a greater proportion of persons in the lowest socioeconomic districts. This socioeconomic gradient could have been underestimated in our study because pet owners in Chile must pay for the **healthcare of their pets, and the likelihood of** diagnostic tests being performed for diseases requiring laboratory confirmation is low, especially in the poorest areas of the city. We also did not account for the overall number of veterinary clinics that exist in each district, making estimation of disease notification rates among districts or health services, impossible. The finding that the most prevalent diseases were preventable by vaccination (e.g., distemper) raises questions about the coverage and quality of vaccinations among pets in Santiago.

The validity of this pilot surveillance system is limited because the overall ANC showed a declining trend during the 2 years of surveillance. This trend probably does not represent reduced incidence of infectious diseases among pets in Santiago; on the contrary, it may illustrate the difficulty of maintaining a private surveillance system based on professional motivation, a key element for ensuring the sustainability of such a system over time.

This pilot surveillance system may motivate other investigations regarding zoonotic infections of pets in Chile. The resulting information would provide the data needed to calculate disease incidence rates and establish unbiased comparisons, which can be used to further the goal of improved pet and human health.
